# Triterpene Acid and Phenolics from Ancient Apples of Friuli Venezia Giulia as Nutraceutical Ingredients: LC-MS Study and In Vitro Activities

**DOI:** 10.3390/molecules24061109

**Published:** 2019-03-20

**Authors:** Stefania Sut, Gokhan Zengin, Filippo Maggi, Mario Malagoli, Stefano Dall’Acqua

**Affiliations:** 1 Department of Agronomy, Food, Natural Resources, Animals and Environment (DAFNAE), Agripolis Campus, University of Padova, 35020 Legnaro (PD), Italy; stefania.sut@studenti.unipd.it (S.S.); mario.malagoli@unipd.it (M.M.); 2 Department of Biology, Science Faculty, Selcuk University, 42250 Konya, Turkey; gokhanzengin@selcuk.edu.tr; 3 School of Pharmacy, Via Sant’Agostino 1, University of Camerino, I-62032 Camerino, Italy; filippo.maggi@unicam.it; 4 Department of Pharmaceutical and Pharmacological Sciences, University of Padova, Via Francesco Marzolo, 5, 35131 Padova (PD), Italy

**Keywords:** ancient apple, triterpene acids, polyphenols, chlorogenic acid, LC-MS, enzyme inhibition

## Abstract

Triterpene acid and phenolic constituents from nine ancient varieties of apple (*Malus domestica*) fruits cultivated in Fanna, Friuli Venezia Giulia region, northeast Italy, were analyzed and compared with four commercial apples (‘Golden Delicious’, ‘Red Delicious’, ‘Granny Smith’ and ‘Royal Gala’). Total phenolic and flavonoid contents were measured by spectrophotometric assays. The quali-quantitative fingerprint of secondary metabolites including triterpene acid was obtained by LC-DAD-(ESI)-MS and LC-(APCI)-MS, respectively. Based on the two LC-MS datasets, multivariate analysis was used to compare the composition of ancient fruit varieties with those of four commercial apples. Significant differences related mainly to the pattern of triterpene acids were found. Pomolic, euscaphyc, maslinic and ursolic acids are the most abundant triterpene in ancient varieties pulps and peels, while ursolic and oleanolic acids were prevalent in the commercial fruits. Also, the content of the phenolic compounds phloretin-2-*O*-xyloglucoside and quercetin-3-*O*-arabinoside was greater in ancient apple varieties. The antioxidant (radical scavenging, reducing power, metal chelating and phosphomolybdenum assays) and enzyme inhibitory effects (against cholinesterase, tyrosinase, amylase and glucosidase) of the samples were investigated in vitro. Antioxidant assays showed that the peels were more active than pulps. However, all the samples exhibited similar enzyme inhibitory effects. Ancient Friuli Venezia Giulia apple cultivars can be a source of chlorogenic acid and various triterpene acids, which are known for their potential anti-inflammatory activity and beneficial effects on lipid and glucose metabolism. Our results make these ancient varieties suitable for the development of new nutraceutical ingredients.

## 1. Introduction

The consumption of fruits and vegetables is very important in human nutrition. They provide nutrients and non-nutritive constituents with significant biological activities, thus contributing to a healthy diet, with reduction of disease risk. Based on scientific data, nutritionists suggest that increasing fruit and vegetable consumption is one of the best strategies to decrease the burden of several chronic diseases. [1] Scientific evidence showed that a higher consumption of fruits and vegetables is associated with a lower risk of all-cause mortality, particularly cardiovascular mortality [2,3,4].

Apples are one of the most consumed and healthy fruits due to their content of high value nutrients and secondary metabolites. Health promoting properties of apple fruit are ascribed to the presence of different groups of polyphenol constituents, namely procyanidins, dihydrochalcones, flavonols and hydroxycinnamic acids that are present in the pulp and are mostly concentrated in the peel [3,5,6,7,8,9,10]. Secondary metabolite composition can be different in apple varieties and the amount of such compounds has been reported to be different of several fold. Other differences can be related to cultivar and pedoclimatic factors [3,5,7,11]. Various attempts were made to optimize the level of healthy compounds in apple fruits through cultivation methods and selection of varieties [2,4,7,8,9,10]. Pulp and peel contents of secondary metabolites are different both from qualitative and quantitative point of views [3,7,10,11,12]. Since the peel is the richest source of phenolics, any promotion of apple consumption should imply this fruit part. Obviously organic cultivation should be preferred in order to avoid the presence of pest residues. There is a large diffusion of the so called ‘nutraceuticals’ as health promoting products and research in this area is proceeding [13] showing the interest of the market and scientists in finding new products. Consumption of natural dietary polyphenols is considered highly positive in promoting cardiovascular health and limiting the effects of cellular aging [4,14]. The apple can be a valuable fruit for the nutraceutical production and its possible uses are related, more than to a specific compound, to its whole phytocomplex [1,3,8,15,16,17,18,19,20].

Dehydration is the most widely used process for preserving foods. Reducing water in apple matrix will slow down, or even inhibit, the microbial and enzymatic activities and extend the shelf-life of the final product. Dried apples may be a significant source of nutritional and bioactive compounds. [21] Administration of dried apples is not ideal in caloric-restricted diets due to the high content of sugars. On the other side, a selective extraction of phytoconstituents would offer the opportunity to administrate a low-calorie product with greater amounts of health-promoting compounds.

Local Italian apple varieties have been considered valuable for the production of nutraceutical ingredients, as in the case of Annurca, a cultivar of Southern Italy [17,22,23,24]. Its extracts are effective on lipid metabolism in HepG2 cell lines [18], and limited the intestinal cholesterol absorption [25]. Procyanidin B2-rich Annurca apple extracts have also cosmeceutical potential in the treatment of alopecia [22]. These findings suggest the potential usefulness of this fruit and the possible exploitation of other unexplored apple varieties for nutraceutical applications.

Apple procyanidins and triterpene acids have been studied for their anti-inflammatory activities [15]. Notably, ursolic acid, which is abundant in apple pomaces [26], is an inhibitor of the inflammatory response, being able to inhibit NF-kB, AP-1 and NF-AT [27]. More recently, ursolic and oleanolic acids have been considered effective for the treatment of type 2 diabetes [28,29].

Apple trees have been grown in different parts of the world for many centuries and farmers used to select numerous varieties, having different sensory and organoleptic properties, ripening time and adaptability to specific soil–climate conditions. Although numerous apple varieties were developed over time, only a few are currently used on a commercial level. As a consequence, several ancient varieties are at risk of extinction despite some favorable traits compared to commercial apples. Thus, their preservation is very important [8,9,14,17,19,30]. The recovery of ancient apple cultivars due to their peculiar composition, taste, resistance to pathogens and adaptability to climate and soil is a challenging task. Ancient apple cultivars are grown in specific regions and in many cases are characterized by limited productions, diffused only to local markets, but interest in their secondary metabolites are growing due to biodiversity conservation and new introduction in the market of such varieties in food markets [9,12,14,17,30,31,32]. Some papers reported analysis of polyphenol composition in ancient varieties [9,17,30]. Previously published papers considered Croatian [30], Polish [8,12,30], and Italian [9,17,33] ancient varieties. The evaluation of the secondary metabolites composition of ancient apple varieties is still limited and can be of interest in term of valorization of such fruits as a source of health-promoting constituents and for nutraceutical applications offering a new market to these overlooked productions.

In this work we considered nine ancient apple varieties preserved and cultivated in Fanna, Friuli Venezia Giulia (FVG) region, Northeast Italy. Literature data about these apple varieties are scarce. Recently, the volatile constituents of some ancient FVG apples were studied in comparison with extensively-cultivated varieties [32], but to our knowledge information on polyphenols and triterpenes content is still limited as well as their bioactivities such as antioxidant and enzyme inhibitory. Here we reported the chemical characterization of triterpene and phenolic of these old apple cultivars by performing Liquid Chromatography Diode Array Electro Spray Mass Spectrometry n-fragmented, LC-DAD-(ESI)-MSn and Liquid Chromatography Atmospheric Chemical Ionization Mass Spectrometry n-fragmented LC-APCI-MSn analyses. Results revealed the secondary metabolites fingerprint of such ancient apples, furthermore in vitro assays evaluating the antioxidant and enzyme-inhibitory activities were performed thus showing their potential application as a source of novel nutraceutical ingredients.

## 2. Results

### 2.1. Secondary Metabolite Fingerprinting of Ancient Apples

**Figure 1:**
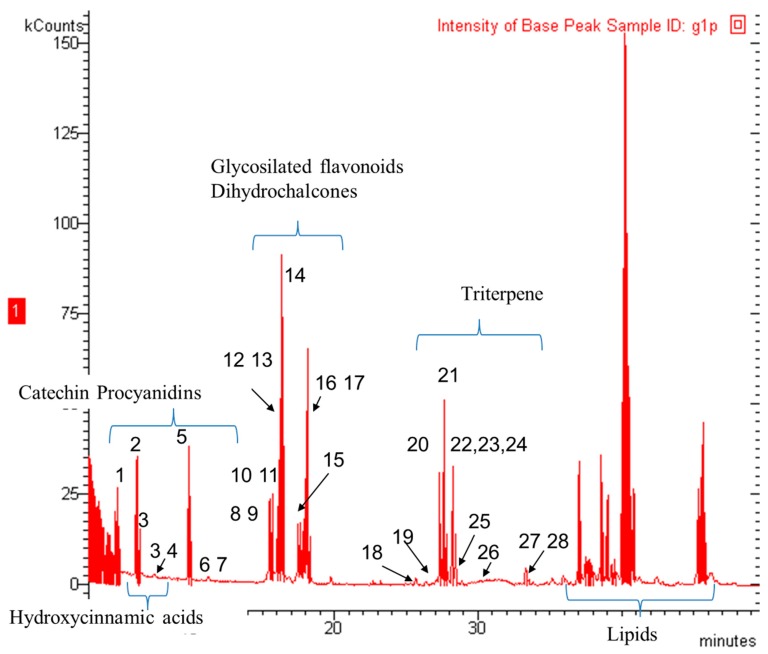
Representative chromatogram of apple peels; the principal classes of phytoconstituents are highlighted; numbers of the identified metabolites are those reported in Table 1.

Fingerprinting of the secondary metabolites of ancient apple samples resulted in the identification of triterpene acids and polyphenols. In Figure 1 a representative LC-ESI chromatogram (total reconstructed current) showing the fingerprint of the analyzed apple peel sample (H1B) with the main classes of constituents, is reported. Compounds were identified on the basis of their retention times, *m*/*z* and collision-induced dissociation spectra plus comparison with authentic standards when available.

**Figure 2:**
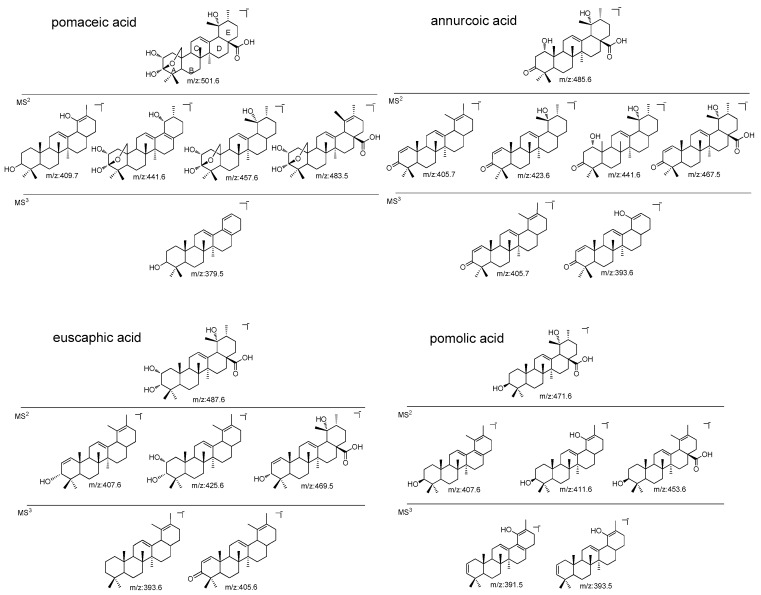
Tentative structures proposed for the main fragment ions of pomaceic, annurcoic, euscaphic and pomolic acids in the MS^n^ study.

As reported in Table 1 the identified apple constituents were summarized according to their main *m*/*z* values and fragments. The main classes of analyzed compounds were triterpene acids, flavan-3-ols and proanthocyanidins (PAC), glycosylated flavonoids, dihydrochalcones, and hydroxycinnamic acids that were detectable in all the samples. Signals ascribable to lipid portions were also detected especially in peel samples. Apple triterpene acids present significant bioactivities and have been up to now less considered compared to polyphenols due to the poor UV absorbtion that in many cases prevent the use of LC-DAD or LC-UV methods. The possibility to rapidly identify the triterpene acids in samples can be useful for assessing differences of the various apples related to these compounds. In a previous paper we reported an LC-APCI method was developed and used to characterize apple triterpene acids. [34] As a continuation of that work, here we present the fragmentation pattern of the main triterpenes detected in FVG ancient apples. Typical collision-induced dissociation spectra are reported in supplementary figures showing the different behavior of structurally related derivatives. The apple triterpene acid namely, pomaceic, annurcoic, euscaphic, pomolic, maslinic, oleanolic, betulinic and ursolic acids presents hydroxyl groups in the ring A and E. Tentatively assigned structures of main fragment ions of pomaceic, annurcoic, euscaphic and pomolic acids are reported in Figure 2 and can offer a starting point for the structure elucidation of such triterpene acids by MS/MS fragmentation. The observed fragments losses are ascribable to water, CO_2_ and methyl groups. At the same time, we observed that up to MS^3^ the pentacyclic triterpene moiety is intact, while several different ion species showed *m*/*z* values that suggest water elimination and formation of isolated or conjugated double bonds. Spectra presented in Supplementary Materials of the various derivatives clearly show the possibility to discriminate several different compounds on the basis of MS^n^ behavior. Derivatives differing only for the methyl groups in position 19 and 20, as oleanolic, ursolic, maslinic and corosolic acids were not discriminated enough up to MS^3^.

The LC-APCI-MS^n^ analysis of apple triterpenes offer the unique opportunity to have structural information on such compounds and to identify the pattern of constituents using low resolution MS^n^ spectrometers. The selectivity of the MS detector represents an improvement in the detection of triterpene acids compared to the proposed methods using (Evaporative Light Scattering Detector) ELSD, UV or diode array detectors, allowing phytochemical screening of different fruits for this class of compounds. The method was also used for quantitative purposes, offering a valuable approach in the quali-quantitative analysis of different apple varieties. In fact, each different derivative was identified on the basis of its [M − H]^−^ and fragmentation pathway, furthermore the triterpene acids amount was measured using oleanolic acid as reference, generally done, for example, for flavonoids or other classes of constituents. The pomaceic acid showed [M − H]^−^ at *m*/*z* 501.6, the MS^2^ spectrum showed signals at *m*/*z* 483.5 (−18 Da) due to water loss, *m*/*z* 457.6 (−44 Da) due to the loss of CO_2_. Further typical ions were at *m*/*z* 441.5 (−60 Da) and 409.7 (−92 Da). In MS^3^ from the specie at *m*/*z* 409.6, a specie at *m*/*z* 379.5 was prevalent. For annurcoic acid the molecular ion [M − H]^−^ was observed at *m*/*z* 485.6, MS^2^ spectrum is characterized by base peak ion at *m*/*z* 423.6 (−62 Da), 467.5 (−18 Da), 441.6 (−44 Da) and 405.7 (−80 Da). These fragments can be generated by water loss, methyl group and carboxyl function but leaving intact the basic structure of the pentacyclic triterpene. The MS^3^ spectrum from the ion at *m*/*z* 423.7 showed the base peak at *m*/*z* 405.7 (−18 Da) and 393.6 (−30 Da). Euscaphyc acid present [M − H]^−^ at *m*/*z* 487.5, in MS^2^ spectrum specie at *m*/*z* 425.6 (−62 Da) ascribable to CO_2_ and water loss, specie at *m*/*z* 469.5 (−18 Da). Further ion at *m*/*z* 407.6 (−80 Da) can be assigned to a further loss of water from *m*/*z* 425.6. MS^3^ from specie at *m*/*z* 425.6 yield the ion at *m*/*z* 405.6 and 393.6 that can be assigned to derivatives presenting the loss of two hydroxyl groups. Pomolic acid present [M − H]^−^ at *m*/*z* 471.6. MS^2^ spectrum present a strong species at *m*/*z* 453.6 (−18 Da), 411.6 (−60 Da) and 407.6 (−64 Da). The MS^3^ fragmentation from 453.6 yield in ions at *m*/*z* 393.5 (−44 Da) and 391.5 (−46 Da).

**Figure 3:**
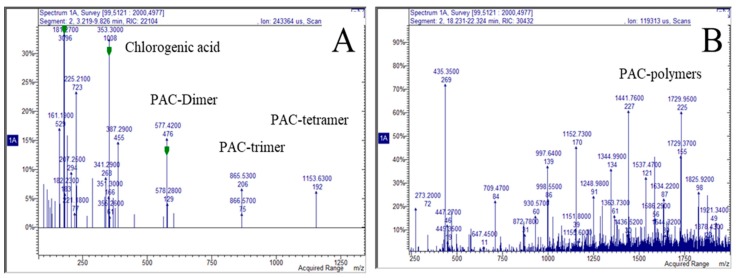
Cumulative spectra obtained from the chromatogram regions 3–10 min (**A**) and 18–22 min (**B**) showing the relative intensity of *m*/*z* species corresponding to different (proanthocyanidin) PAC species.

Pulps of all the samples revealed the presence of procyanidins and the analysis of cumulative MS spectra revealed in the first part of the chromatogram (3–10 min) the presence of oligomeric PAC (dimers up to pentamers) while multiple charged ion species ascribable to polymeric PAC (hexamers to decamers) were detectable in the time range from 18–22 min, representative spectra referring to sample E1P (Figure 3).

### 2.2. Multivariate Analysis

**Figure 4:**
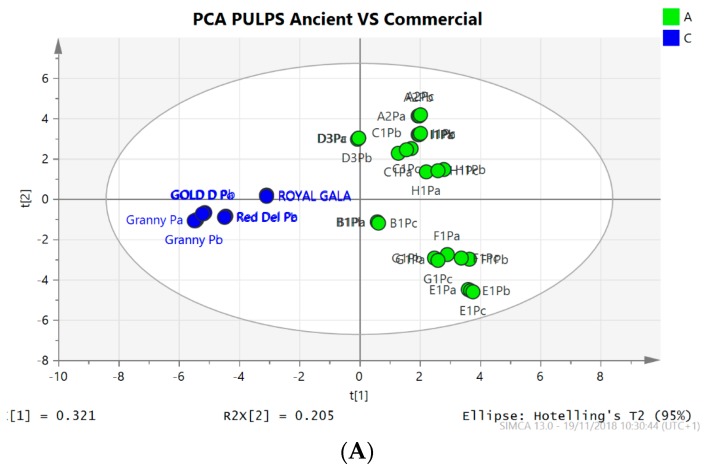

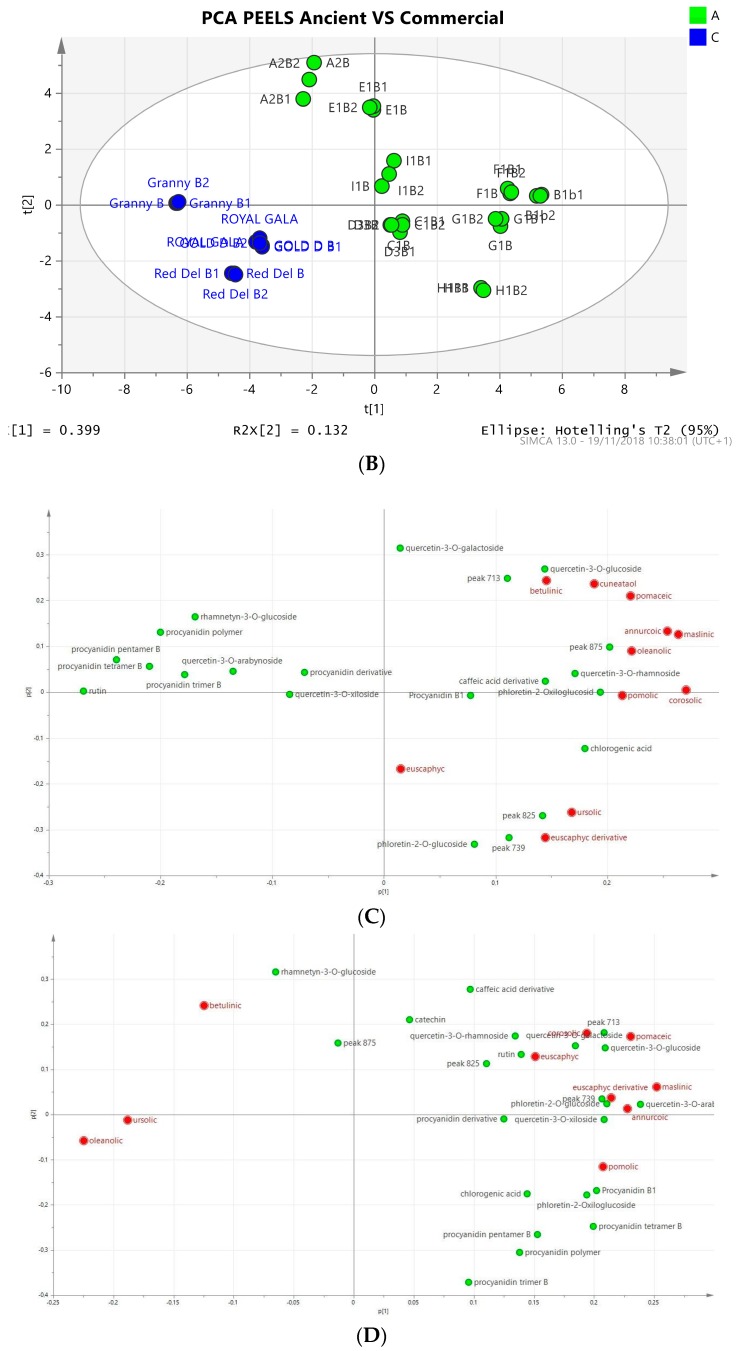
Score scatter and loading plots of the PCA obtained from the LC-MS datasets for pulps (**A**,**C**) and peels (**B**,**D**) of the analyzed apple samples. Commercial (blue) and Ancient (green) samples are highlighted. An evident clustering was observed. Triterpene acids and phenolic compounds are highlighted in the plot with red and green color respectively.

The datasets obtained from the LC-ESI and LC-APCI methods, used for the analysis of polyphenols and triterpene acid respectively, were merged together and targeted multivariate data analysis was performed considering triterpene and phenolic derivatives. Figure 4 summarized the results obtained for four commercial and nine ancient apple varieties. PCA clearly shows differences (R2X 0.321, Q2X 0.2285 for pulps and R2X 0.399, Q2X 0.349 for peels), between the two apple groups indicating that the chemical profiles of peels and pulps of the ancient and commercial apples are different.

Most of the descriptors are significantly discriminant for the ancient fruit pulps and peels. In addition, a different fingerprint of ancient apples was evidenced with respect to commercial ones. Hydroxycinnamic acids and phloretin-2-*O*-xyloglucoside were the discriminant phenolic compounds of fruit pulps, while procyanidins with a larger degree of polymerization characterized the commercial samples.

Triterpene acids had a significant role in discriminating peels of the ancient apples with respect to those of commercial ones. Specific compounds can be observed as discriminant of the two groups in particular for pulps, annurcoic, maslinic, pomolic, pomaceic, corosolic acid and oleanolic are present in the ancient apple while they are not detectable in the commercial pulp samples. Phloretin-2-*O*-xylogucoside peak area is increased in ancient varieties (+35%) while rutin is higher in commercial fruits pulps (+112%) compared to the average area values of each compound in total samples. Related to peels, ancient varieties are characterized by maslinic acid (+44 %), annurcoic acid (+34%), pomaceic acid (not detectable in commercial samples) and quercetin-3-*O*-arabinoside (+24%) while commercial apple present incremented amounts of oleanolic acid (+174%), and ursolic acid (+175%) compared to ancient varieties.

### 2.3. Spectrophotometric Assays to Assess Total Phenolic and Flavonoid Contents

**Figure 5:**
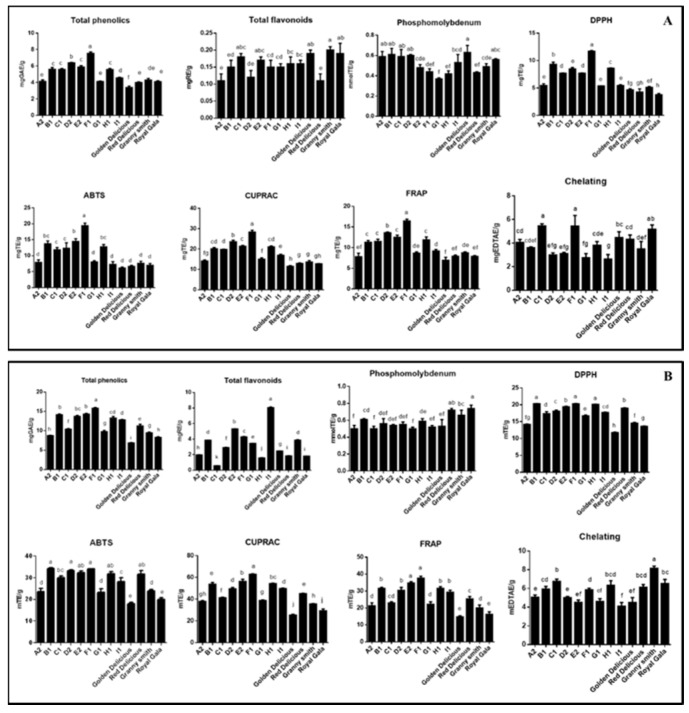
Total phenolic (TPC), total flavonoid (TFC) and antioxidant properties of tested Pulp extracts (**A**) and Peel extracts (**B**). Values expressed are means ± S.D. of three parallel measurements. GAE: Gallic acid equivalent; RE: Rutin equivalent; TE: Trolox equivalent; EDTAE: EDTA equivalent. Different letters indicated significant differences in each group (pulp or peels) (*p* < 0.05).

The total phenolic and flavonoid contents of pulps and peels samples were detected by spectrophotometric assays. Generally, peels had higher concentration of these compounds as compared to pulps (Figure 5). The highest levels of phenolic and flavonoids were detected in F1B (15.83 mgGAE g^−1^) and I2B (8.05 mgRE g^−1^), respectively. Such preliminary data are useful to have a rapid comparison of all the samples and to assess any possible relation also with antioxidant results. To assess a more accurate variation in the quali-quantitative profile of the apple samples, quantitative data on phenolics were also obtained using LC-DAD-ESI to avoid any interference from other apple constituents.

### 2.4. Pulp Composition

**Figure 6:**
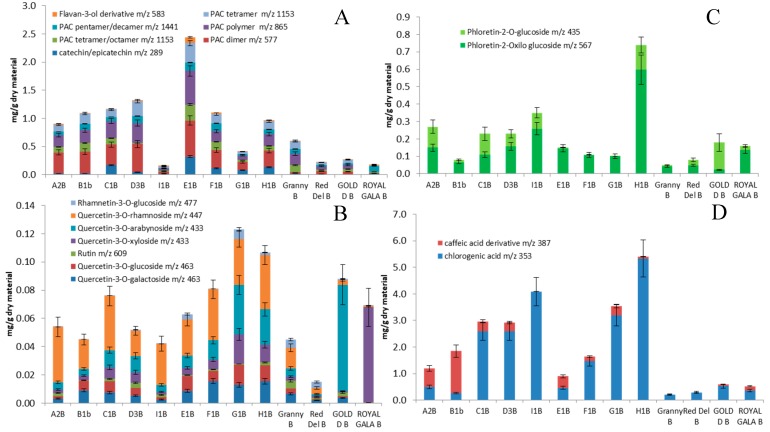

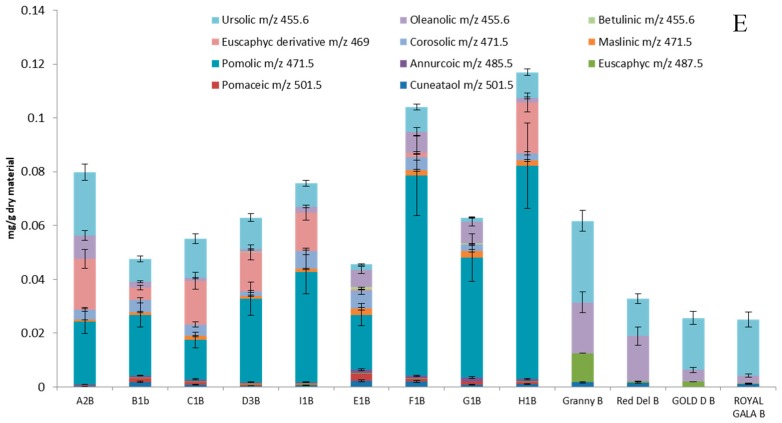
Contents, mg/g on dry material, of flavan-3-ols and procyanidins (**A**), flavonoids (**B**), chalcones (**C**), hydroxycinnamic acids (**D**) and triterpene acids (**E**) in apple pulps. In the *X* axis: Granny: ‘Granny Smith’, Red Del: ‘Red Delicious’, Gold D: ‘Gold Delicious’.

**Figure 7:**
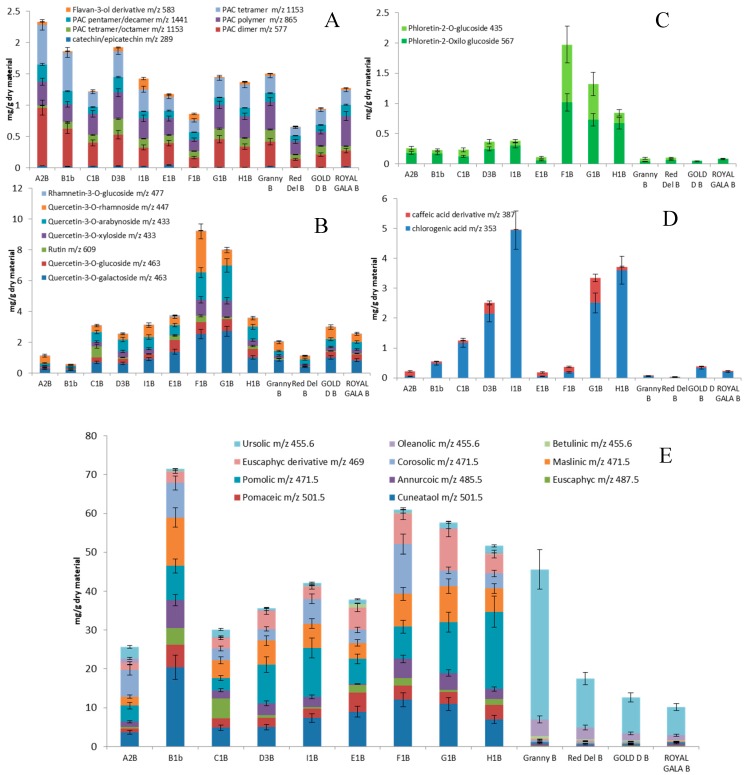
Contents, mg/g on dry material, of flavan-3-ols and procyanidins (**A**), flavonoids (**B**), chalcones (**C**), hydroxycinnamic acids (**D**) and triterpene acids (**E**) in apple peels. In the X axis: Granny: ‘Granny Smith’, Red Del: ‘Red Delicious’, Gold D: ‘Gold Delicious’.

Triterpene acid was present in low amounts compared to peels (*p* < 0.05) for both groups. The average values were 0.0723 and 0.0363 mg/g for ancient and commercial samples, respectively. Significant differences (*p* < 0.05) were observed with the ancient varieties being richer in pomolic, pomaceic, maslinic and annurcoic acids than commercials. Higher amounts of ursolic and oleanolic acids were found in commercial apples, representing the 79–95% of total triterpene content. Average PAC and catechin derivatives values in pulps were significantly different (*p* < 0.05) between ancient (1.06 ± 0.64 mg g^−1^) and commercial (32 ± 0.19 mg g^−1^) varieties (Figure 6). Total PAC in E1P was of 2.44 mg/g, two- and three-folds higher than the average of ancient and commercial samples. In all the ancient apple pulps, PAC dimers (*m*/*z* 577) were the most abundant compounds of PAC ranging from 24–38% of total PAC content. In the commercial samples they ranged from 3–24% of total PAC content. Alike, the concentration of caffeic acid derivatives in ancient apples were significantly (*p* < 0.05) higher (2.70 mg g^−1^) than in commercial samples (0.41 mg g^−1^). Sample H1P containing 5.4 mg/g of caffeic acid derivatives was thirteen-fold higher than the commercial samples. The average values of the flavonoid content did not differ significantly between the two groups of samples with average values of 0.071 and 0.055 mg/g, respectively. Notably, samples G1P and H1P showed the highest amounts, 0.123 and 0.107 mg/g dry material, respectively (Figure 7). Quercetin-3-*O*-rhamnoside accounted for 26–78% of the total flavonoid content in ancient apples. Different quercetin derivatives dominated the total flavonoid content of commercial samples, with quercetin-3-*O*-xyloside as the most abundant (97%) in ‘Royal Gala’, and quercetin-3-*O*-arabinoside (85%) in ‘Golden Delicious’. Two dihydrochalcones, namely phloretin-2-*O*-xyloglucoside and phloretin-2-*O*-glucoside were found in ancient apple samples with average values of 0.251 and 0.118 mg g^−1^, and the average value of dihydrocalchone was significantly higher (*p* < 0.05) than commercial varieties. Sample H1P showed the highest amount of these dihydrochalcones with a concentration value of 0.74 mg/g. On the other hand, the varieties E1P, F1P and G1P contained mainly phloretin-2-*O*-xyloglucoside. Among commercial varieties, ‘Golden Delicious’ pulps showed phloretin-2-*O*-glucoside accounting for 88% of total chalcones.

### 2.5. Peels Composition

The triterpene content in ancient apple peels (45.9 mg g^−1^) doubled that of commercial ones (21.5 mg g^−1^) being significantly higher (*p* < 0.05) for all the ancient varieties compared to the four commercial ones. As for pulp samples, a different pattern of constituents was observed. Cuneataol, pomaceic, annurcoic, maslinic, pomolic and corosolic acids represented the most abundant derivatives in ancient apples, whereas ursolic and oleanolic acids were the predominant compounds in commercial samples. This suggests the possibility to consider triterpene acids as marker compounds to check the authenticity of such ancient varieties.

Catechin, PAC and flavonoid contents of peels of ancient and commercial samples were in the same concentration range both from a qualitative and quantitative point of view. Procyanidin dimer (*m*/*z* 577) represented the 18–40% of total PAC content in ancient varieties (19–26% in commercial ones). Average values of PAC derivatives in ancient apple peels were higher than those of commercial apples, being 1.51 and 1.09 mg g^−1^, respectively (Figure 7). Average values of caffeic acid derivatives were 1.90 and 0.18 mg/g for ancient and commercial samples, respectively. However, a high variation was observed in the various ancient varieties, with concentrations ranging from 0.19 (E1B) to 4.97 mg/g (I1B). The average amount of flavonoids was similar in the two apple groups being 3.86 and 2.16 mg/g for ancient and commercial samples, respectively. Quercetin-3-*O*-galactoside was the most abundant flavonoid derivative in the four commercial samples and in five out of nine ancient apple varieties. Other abundant flavonoids in ancient apples were rutin (C1B), quercetin-3-*O*-arabinoside (D3B) and quercetin-3-*O*-rhamnoside (A2B and F1B).

### 2.6. Biological Activities

To provide a full picture for antioxidant properties of the apple samples, different in vitro assays (phosphomolybdenum, metal chelating, reducing power (CUPRAC and FRAP) and radical quenching (DPPH and ABTS)) were performed, data are reported in Figure S5 and Tables S1–S3 in Supplementary Materials. In phosphomolybdenum assay, the samples exhibited similar activities and the best activity was observed in peels of ‘Royal Gala’ (0.74 mmolTE/g), followed by peels of ‘Red delicious’ (0.72 mmolTE/g) and ‘Granny Smith’ (0.66 mmolTE/g) (Figure 5). In previous papers [35,36,37], the assay was used to assess total antioxidant capacity of extracts. In reducing power assays, F1B exhibited the strongest ability (62.72 mgTE/g for CUPRAC and 37.66 mgTE/g for FRAP), followed by E2B, H1B and B1B (Figure 5). These results seem to be linked to the fact that these extracts had a higher concentration of total phenolics and flavonoids. This approach was also confirmed by several researchers, who reported a linear correlation between total phenolic and reducing abilities. [38,39]. Regarding radical quenching assays (ABTS and DPPH), B1B and F1B had significant higher activity than other samples (Figure 5). Similar to total bioactive compounds, peel samples were more active on these radicals than pulp samples. In this sense, observed activity could be attributed to levels of total bioactive compounds and this is accordance with earlier reports [40,41].

Chelating ability of transition metal is another important mechanism and this is closely related to production of hydroxyl radical, which is the most dangerous radical. The potent metal chelating abilities were found to be in the peel of C1 (6.76 mgEDTAE/g), ‘Royal Gala’ (6.53 mgEDTAE/g) and H1 (6.34 mgEDTAE/g). These observation differed for radical scavenging and reducing power assays. This fact could be explained with the presence of non-phenolic chelators such as polysaccharides, or peptides in the extracts [42,43].

**Figure 8:**
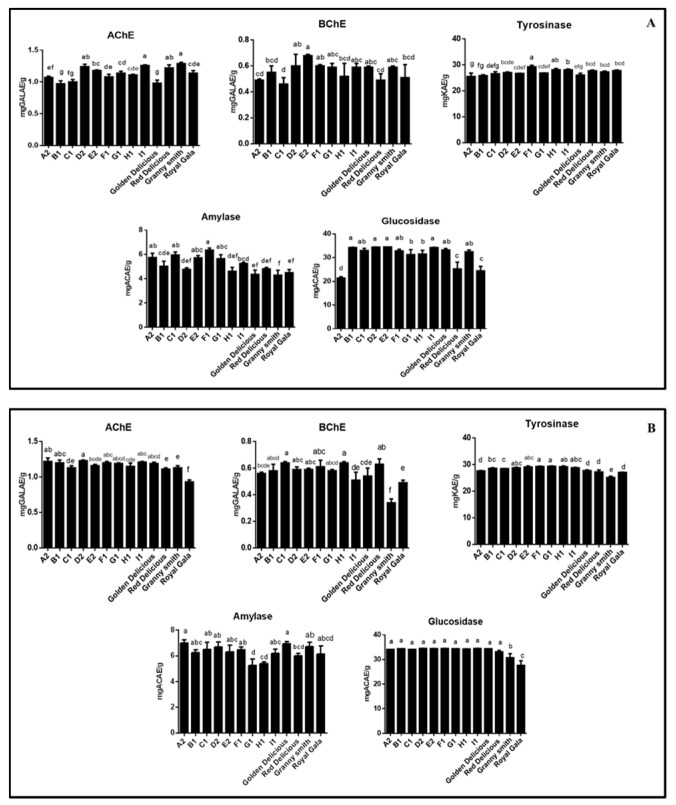
Enzyme inhibitory properties of tested Pulp extracts (**A**) and Peel extracts (**B**). Values expressed are means ± S.D. of three parallel measurements. GALAE: Galatamine equivalent; KAE: Kojic acid equivalent; ACAE: Acarbose equivalent. Different letters indicated significant differences in each group (pulp or peels) (*p* < 0.05).

The enzyme inhibitory capacity of the apple samples was investigated towards cholinesterases, tyrosinase, amylase and glucosidase due to the very well-known importance of such enzymes in some pathological diseases (Figure 8). The pulps and peels had similar AChE and BChE inhibitory effects. The strongest abilities was observed in the pulp of ‘Granny smith’ (1.29 mgGALAE/g for AChE) and E2P (0.68 mgGALAE/g for BChE), respectively. Additionally, G1B (29.35 mgKAE/g) and F1B (29.27 mgKAE/g) showed very close tyrosinase inhibitory effects. A similar case was found for A2B (6.99 mgACAE/g) and the pulp of ‘Golden delicious’ (6.94 mgACAE/g) against amylase. The most potent glucosidase inhibitory effects were found in E2P, D2B and I1B and their results were similar. Generally, the peel samples had higher inhibitory effects when compared to pulp samples against the tested enzymes. From this point, the presence of proanthocyanidins, triterpenoids or hydroxycinnamic acids might be attributed to observed enzyme inhibitory effects. These compounds have been already reported as anti-Alzheimer and anti-diabetic agents [44,45,46,47,48].

## 3. Discussion

Apples are one of the most important fruits in the human diet and in the last thirty years the market has moved towards modern cultivar due to organoleptic and yield issues. For these reasons, in many countries, the ancient cultivars have been considered obsolete and replaced, leading to a loss of fruit biodiversity. Nevertheless in the context of renewed consumer interest in locally produced foods, the ancient varieties are now being re-considered. Another aspect is related to environmentally acceptable cultivar practice, and these ancient varieties posses also particular traits of resistance to climate and pathogens. The safeguard of ancient apple varieties appears relevant for the possible exploitation in the food market given the specific organoleptic qualities. But an attractive opportunity to valorize such ancient fruits is to develop their use for nutraceutical purposes, thanks to the high content in health promoting secondary metabolites.

In this context the results obtained in the present paper report for the first time the characterization of nine ancient apple varieties of Friuli Venezia Giulia region. The triterpene acid content result was different than the compared conventional varieties and should be taken into account for the possible use as nutraceutical. In fact, different papers reported the beneficial effects of triterpene acids on glucose metabolism with possible application in glycemic control [28,29]. Oleanolic acid has a potential role in treating drug-induced hepatic steatosis due to its interaction with liver X receptor alpha and pregnane X receptor reducing ligand-induced lipogenesis [49]. In addition, it is well known that different triterpene acids from various natural sources present anti-inflammatory effects. For instance, recent papers showed significant effects of maslinic acid in inflammation [50] and explained part of its activity by NF-κB inactivation [51]. Anti-inflammatory and anti-arthritic effects through NF-κB inactivation were reported for maslinic and pomolic acids [50]. Ancient FVG apple varieties are significant sources of such compounds and further investigations are needed to assess potential anti-inflammatories and glycemic control effects of their constituents.

Significantly higher levels of dihydrochalcones (*p* < 0.05) and hydroxycinnamic acids (*p* < 0.05) were measured in peels and pulps of the ancient varieties compared to the commercial ones.

The phenolic composition of the pulp, which represents more than the 95% of the total fruit dry weight, is especially rich in chlorogenic acid and, for the samples H1P, G1P and I1P, in procyanidins. Chlorogenic acid has pronounced effects on glucose and lipid metabolism [49] being considered useful for weight management and obesity. Green coffee beans are in general used for the production of nutraceutical ingredients as they contain chlorogenic acid ranging from 3.5–14% (*w/w* dry matter) [52]. Among the ancient cultivars, the highest chlorogenic acid contents (0.4–0.5% *w/w* dry matter) were found in the pulp of I1 and H1 fruits. Apples of the ancient cultivars can be thus considered as a good source of chlorogenic acid offering a different source than green coffee with the advantage of the absence of caffeine and trigonelline.

Preliminary in vitro assays revealed significant antioxidant and inhibitory enzyme activities showing potential usefulness. These data showed the effects on enzymes that are involved in metabolic and degenerative diseases and indicate the need for further investigations into the possible usefulness of such compounds related to Alzheimer’s disease, hyperpigmentation and type 2 diabetes. The observed biological effects could be attributed to the phytochemicals in these extracts, such as triterpenoids, proanthocyanidins and hydroxycinnamic acids. Thus, the ancient apples could be regarded as a valuable source for biologically-active compounds in view of nutraceuticals. LC-MS^n^ analysis with different ion sources allowed the identification of secondary metabolites yielding an accurate fingerprinting of these fruits. Ancient FVG apple cultivars can be a source of chlorogenic acid and different triterpene acids, which have been studied in literature for their potential anti-inflammatory activity and beneficial effects on lipid and glucose metabolism.

## 4. Materials and Methods

### 4.1. Plant Materials and Sampling

Apples of nine ancient varieties (10 kg each) were collected with the assistance of Associazione Amatori Mele Antiche (Pordenone, Fanna, Friuli Venezia Giulia, Italy, N 46°11′; E 12°45′, 274 m a.s.l.). Fruits were washed and peels and pulps separately cut in thin slices. Samples indicated with “B” and “P” letters refer to peels and pulps, respectively. Sliced peels and pulps were put into a ventilated oven at 55 °C and dried up to constant weight. Samples were then grinded, transferred into Eppendorf tubes (50 mL) and stored at −20 °C until sample preparation and analysis. The nine varieties were indicated with letters as follows: Di Corone A2; Rosso Invernale B1; Limoncello antiche C1; Pomacia D2; De la rosa E1; Belladonna F1; Regina G1; Del Mieli H1; Di Giulio I1. Commercial samples of ‘Golden Delicious’, ‘Red Delicious’, ‘Granny Smith’ and ‘Royal Gala’ varieties were bought in local stores of Padova, Italy. Samples of peels and pulps were indicated for all the apples varieties with letter P and B respectively. A voucher sample of each apple dried material (peels and pulps) is stored at the Department of Pharmaceutical and Pharmacological Sciences of University of Padova.

### 4.2. Chemicals and Instruments

Methanol, acetonitrile, formic acid, rutin, phloridzin and catechin were obtained from Sigma-Aldrich (Milan, Italy). Procyanidin B2, oleanolic acid, asiatic acid were obtained from Extrasynthese (Genay Cedex, France). An Agilent Eclipse XDB C-18 3.0 × 150 mm (3.5 µm) was used as stationary phase for analytical procedures. For semipreparative HPLC separation of triterpene acids, an Agilent Eclipse C-18 30 × 210 mm (5 µm) stationary phase was used. An Agilent 1100 system equipped with 1100 series diode array, Sedex 60 Evaporative Light Scattering Detector (ELSD) and Agilent 1260 series fraction collector was used for purification of triterpene acids. For LC-DAD-MS analysis an Agilent 1260 chromatograph equipped with 1260 series diode array was used (Agilent, Santa Clara, CA, USA). After the chromatography column a “T” junction split the flow to DAD and Varian MS 500 mass spectrometer (Varian, Santa Clara, CA, USA). MS spectra were acquired using two different approaches, one using Electrospray (ESI) and one Atmospheric Pressure Chemical Ionization (APCI) sources for polyphenols and triterpenes, respectively. For both analyses the spectrometer operated in negative ion mode acquiring spectra in the range *m*/*z* 50–2000.

### 4.3. Sample Preparation

Dried pulp and peel samples were weighed in triplicate (500 mg ± 0.1 mg) and transferred in flasks. Fifteen mL of methanol–water (50%) solution was added and ultrasound assisted extraction was performed at room temperature for 15 min. Liquid was decanted and filtered; further 5 mL of solvent was added to solid material and sonicated for further 15 min. The supernatants were combined and volume adjusted to 25 mL with the same solvent. Samples were then filtered through 0.45 µm membrane filters and used for analysis.

### 4.4. HPLC-DAD-(ESI)-MS Analysis

Separation of polyphenols was achieved on an Agilent Eclipse XDB C-18 (3.0 × 150 mm) 3.5 μm eluting with acetonitrile (A) and H_2_O 0.1% formic acid (B). A gradient program was used as follows: 0 → 15th min: A:B (5:95) → A:B (15:85) 15 → 35th min: A:B (85:15) → A:B (100:0) 48 → 53th min: A:B(100:0) → A:B (5:95). After the chromatography column a “T” junction split the flow to DAD and Varian MS 500 mass spectrometer with ESI as ion source. Flow rate was set at 500 µL/min. For qualitative purposes, MS^n^ spectra was used for identification of the compound, with the use of reference when available. For quantification, DAD detector was set at 280, 330 and 350 nm. Compounds were identified on the basis of MS data and UV spectra and classified in four classes namely proanthocyanidins, dihydrochalcones, hydroxycinnamic acids and flavonols. For quantification of these classes of compounds the following reference compounds were used namely catechin, phloridzin, chlorogeinc acid and rutin. Standard solutions were prepared for the calibration curves in the concentration range of 5–100 µg/mL: catechin at 280 nm, y = 20.525x + 3.2962 (r^2^ = 0.999); phloridzin at 280 nm, y = 87.029x – 1.832 (r^2^ = 0.9998); chlorogenic acid at 330 nm, y = 47.359x + 439.99 (r^2^ = 0.9951); rutin at 350 nm, y = 27.788x + 330.7 (r^2^ = 0.9981).

### 4.5. HPLC-(APCI)-MS Analysis

Triterpenes were separated on an Agilent Eclipse XDB C-18 (3.0 × 150 mm) 3.5 μm using methanol (A) and H_2_O (B) as mobile phases. A gradient program was used as follows: 0 → 12th min: A:B (45:55) → A:B (80:20) 12 → 48th min: A:B (80:20) → A:B (80:20) 48 → 49th min: A:B (80:20) → A:B (45:55) 49 → 55th min: A:B (45:55) → A:B (45:55). Flow rate was 500 µL/min. After the chromatography column, a Varian MS 500 mass spectrometer was used using APCI as ion source. Identification of triterpene was done on the base of MS^n^ spectra and NMR spectra of isolated compounds as reported in our previous work. [34]. For quantification purposes we adopted oleanolic acid as the reference standard and the calibration curve was obtained in the range 5–150 µg/mL, y = 18755x + 961.31 (r^2^ = 0.9992). Quantification was obtained using [M − H]^−^ of each triterpene acid referring to oleanolic acid calibration.

### 4.6. Multivariate Analysis

Data modelling was performed by applying the multivariate technique based on projection. Specifically, principal component analysis (PCA) was used for exploratory data analysis and for highlighting differences of composition between the various grouped samples. Matrix was created merging data obtained by LC-DAD-ESI-MS for the phenolic constituents as well as the LC-APCI-MS for the triterpene acids. Twenty-eight different compounds were used as descriptors of the ancient and commercial apple samples. The data matrix was imported in SIMCA 12 (Umetrics, Sweden) and used to perform PCA analysis. Significant variation of the area of the considered compounds in ancient versus commercial samples were calculated using ANOVA (*p* < 0.05).

### 4.7. Total Bioactive Compounds

With reference to our previous studies [35], the total amount of phenolics (by standard Folin–Ciocalteu method) and flavonoids (by AlCl_3_ method) were determined. Standard compounds (gallic acid (mg GAE/g) for TPC and rutin (mg RE/g) for TFC, respectively) were used to express the obtained results.

### 4.8. Assays for Enzyme Inhibition and Antioxidant Capacity

Tyrosinase, α-amylase, α-glucosidase and cholinesterases (acetylcholinesterase (AChE) and butyrlcholinesterase (BChE)) were selected as the target enzyme and the procedures of these assays were described in our earlier work [35]. Standard inhibitors (acarbose (for α-amylase and α-glucosidase), galantamine (for AChE and BChE), and kojic acid (for tyrosinase)) were used to express the enzyme inhibitor properties.

The antioxidant capacity of the extracts was spectrophotometrically screened by different experiments, namely the ferrozine assay (for chelating abilities), phosphomolybdenum, reduction potentials (by FRAP and CUPRAC assays) and radical scavenging activity (using DPPH and ABTS radicals). Standard compounds (TE/g and EDTAE/g) were used to express the antioxidant properties. The procedures of assays were given reported in our earlier work [35].

### 4.9. Statistical Evaluation for Total Bioactive Compounds and Biological Activity Assays

All tests were carried out in triplicates and findings are expressed as mean value ± SD. One-way ANOVA followed by Tukey’s multiple range was done to investigate significant differences (*p* < 0.05) between the tested samples. SPSS v. 17.0 (SPSS Institute Inc., Cary, NC, USA) was employed for statistical analysis.

## 5. Conclusions

The results presented related to the ancient apple fruits of FVG show their phytochemical composition and in vitro bioactivities offering a starting point for these fruits to be exploited as vegetal material for the production of new nutraceutical ingredients. The valorization of this varietal patrimony by nutraceutical application can offer new profitable opportunities for their cultivation and strengthen the preservation of local biodiversity.

## Figures and Tables

**Table 1:** The identified apple constituents according to their main *m*/*z* values and fragments; * compared with reference standards; ns: not significant.

n.	RT (min)	UV (nm)	[M − H]^−^	MS^2^	MS^3^ and MS^4^	Compound
1	5	280	289	245–205–179		catechin *
2	6.4	330	353	191–179	173–171–127–85	chlorogenic acid *
3	6.5 and 6.8	280	577	451–425–407–289	407–381	procyanidin dimer B1 *
4	6.9	330	387	341–179–161–143		caffeic acid derivative
5	9.8	280	865	739–713–695–577–425–407		procyanidin trimer B
6	11.1	280	1441	1151–865–577		procyanidin pentamer B
7	11.9	280	1153	1027–983–865–577–575		procyanidin tetramer B
8	14.9	280	583	289		flavan-3-ol derivative
9	15	350	463	301	271–255–179	Quercetin-3-*O*-galactoside *
10	15.5	350	463	301	271–255–179	quercetin-3-*O*-glucoside *
11	15.5	350	609	301	271–255–179	rutin *
12	16.1	350	433	301	271–255–179	quercetin-3-*O*-xyloside *
13	16.5	280	567	273	167	phloretin-2-*O*-xyloglucoside *
14	16.9	350	433	301	271–255–179	quercetin-3-*O*-arabynoside *
15	17.2	350	447	301	271–255–179	quercetin-3-*O*-rhamnoside *
16	18.2	280	435	273	167–123	phloretin-2-*O*-glucoside *
17	18.5	350	477	315–285–274		rhamnetin-3-*O*-glucoside *
18	25.5	ns	501.5	471.6–453.6–427.6–409.6		cuneataol
19	26.1	ns	501.5	483.5–457.6–441.5–409.7	379.5	pomaceic acid *
20	27.1	ns	487.5	469.5–425.6–407.6	405.6–393.6	euscaphyc acid *
21	28	ns	485.6	467.5–441.6–423.6–405.7	405.7–393.6	annurcoic acid *
22	28.5	ns	471.6	453.6–411.6–407.6	393.5–391.5	pomolic acid *
23	28.6	ns	471.5	423.5–405.6–393.5–377.6		maslinic acid *
24	28.7	ns	471.5	451.6–423.5–405.6–393.5	407.5–405.6–393.5	corosolic acid *
25	29.5	ns	469.5	423.6–405.5–393.6		euscaphic acid derivative
26	30.5	ns	455.5	414.7–409.7–393.6		betulinic acid *
27	33	ns	455.5	407	391.5–378.6–365.5	oleanolic acid *
28	33	ns	455.5	407	391.5–378.6–365.5	ursolic acid *

## Supplementary Materials

Supplementary materials report tables with bioactivity of extracts and MS fragmentation spectra of triterpene acids.
