# Supplementary material for: Triterpene Acid and Phenolics from Ancient Apples of Friuli Venezia Giulia as Nutraceutical Ingredients: LC-MS Study and In Vitro Activities
Source: Molecules. 2019 Mar 20;24(6):1109. doi: 10.3390/molecules24061109 (PMC6471914; doi:10.3390/molecules24061109)
Supplement: Supplementary file 1 [file molecules-24-01109-s001.pdf]

**Triterpene acid and phenolics from Ancient apples of Friuli Venezia Giulia as nutraceutical ingredients: LC-MS study and *in vitro* activities**

*Stefania Sut*[a], *Gokhan Zengin*[b], *Filippo Maggi*[c], *Mario Malagoli*[a], *Stefano Dall'Acqua*[d*]

[a] Department of Agronomy, Food, Natural Resources, Animals and Environment (DAFNAE), Agripolis Campus, University of Padova, 35020 Legnaro (PD), Italy stefania.sut@studenti.unipd.it, mario.malagoli@unipd.it

[b] Department of Biology, Science Faculty, Selcuk University, Campus, Konya, Turkey gokhanzengin@selcuk.edu.tr

[c] School of Pharmacy, Via Sant'Agostino 1, I-62032 Camerino, University of Camerino Italy; filippo.maggi@unicam.it

[d] Department of Pharmaceutical and Pharmacological Sciences, University of Padova, Via Francesco Marzolo, 5, 35131 Padova (PD), Italy stefano.dallacqua@unipd.it

Table 1. Total bioactive components and total antioxidant capacity (by phosphomolybdenum) of apple samples

| | Samples | Total phenolic content (mgGAE/g) | Total flavonoid content (mgRE/g) | Phosphomolybdenum (mmolTE/g) |
|---|---|---|---|---|
| **Pulp** | *A2* | 4.13±0.18$^{e*}$ | 0.11±0.02$^{e}$ | 0.59±0.05$^{ab}$ |
| | *B1* | 5.60±0.21$^{c}$ | 0.15±0.02$^{cd}$ | 0.61±0.06$^{ab}$ |
| | *C1* | 5.58±0.13$^{c}$ | 0.18±0.01$^{abc}$ | 0.59±0.07$^{ab}$ |
| | *D2* | 6.38±0.06$^{b}$ | 0.12±0.02$^{de}$ | 0.60±0.01$^{ab}$ |
| | *E2* | 5.87±0.18$^{c}$ | 0.17±0.01$^{abc}$ | 0.48±0.03$^{cde}$ |
| | *F1* | 7.54±0.17$^{a}$ | 0.15±0.02$^{cd}$ | 0.44±0.03$^{def}$ |
| | *G1* | 4.12±0.07$^{e}$ | 0.15±0.01$^{cd}$ | 0.37±0.01$^{f}$ |
| | *H1* | 5.57±0.14$^{c}$ | 0.16±0.02$^{bc}$ | 0.42±0.03$^{ef}$ |
| | *I1* | 4.55±0.08$^{d}$ | 0.16±0.01$^{bc}$ | 0.53±0.08$^{bcd}$ |
| | *Golden Delicious* | 3.38±0.06$^{f}$ | 0.19±0.01$^{ab}$ | 0.63±0.07$^{a}$ |
| | *Red Delicious* | 3.99±0.09$^{e}$ | 0.11±0.02$^{e}$ | 0.43±0.01$^{ef}$ |
| | *Granny smith* | 4.29±0.20$^{de}$ | 0.20±0.01$^{a}$ | 0.49±0.03$^{cde}$ |
| | *Royal Gala* | 4.10±0.12$^{e}$ | 0.19±0.03$^{ab}$ | 0.56±0.01$^{abc}$ |
| **Peels** | *A2* | 8.77±0.08$^{h}$ | 1.97±0.02$^{h}$ | 0.50±0.04$^{f}$ |
| | *B1* | 14.13±0.21$^{b}$ | 3.86±0.01$^{d}$ | 0.61±0.01$^{cd}$ |
| | *C1* | 10.41±0.19$^{f}$ | 0.58±0.01$^{k}$ | 0.50±0.03$^{f}$ |
| | *D2* | 13.71±0.28$^{bc}$ | 2.91±0.03$^{f}$ | 0.56±0.06$^{def}$ |
| | *E2* | 14.29±0.22$^{b}$ | 5.31±0.03$^{b}$ | 0.54±0.01$^{def}$ |
| | *F1* | 15.83±0.19$^{a}$ | 4.29±0.06$^{c}$ | 0.55±0.03$^{def}$ |
| | *G1* | 9.75±0.34$^{g}$ | 3.45±0.02$^{e}$ | 0.50±0.02$^{f}$ |
| | *H1* | 13.30±0.39$^{cd}$ | 1.59±0.01$^{j}$ | 0.59±0.03$^{de}$ |
| | *I1* | 12.79±0.14$^{d}$ | 8.05±0.09$^{a}$ | 0.52±0.02$^{ef}$ |
| | *Golden Delicious* | 6.87±0.10$^{i}$ | 2.47±0.02$^{g}$ | 0.53±0.04$^{ef}$ |
| | *Red Delicious* | 11.26±0.44$^{e}$ | 1.84±0.04$^{i}$ | 0.72±0.02$^{ab}$ |
| | *Granny smith* | 9.49±0.18$^{g}$ | 3.86±0.07$^{d}$ | 0.66±0.06$^{bc}$ |
| | *Royal Gala* | 8.27±0.18$^{h}$ | 1.81±0.01$^{i}$ | 0.74±0.04$^{a}$ |

[*] Values expressed are means ± S.D. of three parallel measurements. GAE: Gallic acid equivalent; RE: Rutin equivalent. Different letters indicated significant differences in each group (pulp or peels) ($p < 0.05$).

Table 2. Antioxidant properties of the apple samples

| | Samples | DPPH (mgTE/g) | ABTS (mgTE/g) | CUPRAC (mgTE/g) | FRAP (mgTE/g) | Metal chelating (mgEDTA/g) |
|---|---|---|---|---|---|---|
| **Pulp** | *A2* | $5.45 \pm 0.33^{e*}$ | $7.89 \pm 0.81^{d}$ | $14.15 \pm 0.49^{fg}$ | $7.78 \pm 0.90^{ef}$ | $4.05 \pm 0.26^{cd}$ |
| | *B1* | $9.32 \pm 0.39^{b}$ | $13.73 \pm 0.92^{bc}$ | $20.14 \pm 0.62^{cd}$ | $11.25 \pm 47^{c}$ | $3.61 \pm 0.07^{cdef}$ |
| | *C1* | $7.70 \pm 0.03^{d}$ | $11.83 \pm 0.75^{c}$ | $19.77 \pm 0.18^{d}$ | $11.47 \pm 0.55^{c}$ | $5.46 \pm 0.18^{a}$ |
| | *D2* | $8.48 \pm 0.24^{e}$ | $12.34 \pm 1.72^{c}$ | $23.55 \pm 0.79^{b}$ | $13.59 \pm 0.17^{b}$ | $2.99 \pm 0.18^{efg}$ |
| | *E2* | $7.69 \pm 0.04^{d}$ | $14.48 \pm 0.84^{b}$ | $21.40 \pm 0.41^{c}$ | $12.47 \pm 0.53^{bc}$ | $3.11 \pm 0.10^{efg}$ |
| | *F1* | $11.71 \pm 0.11^{a}$ | $19.42 \pm 0.82^{a}$ | $28.32 \pm 0.92^{a}$ | $16.44 \pm 0.44^{a}$ | $5.44 \pm 0.90^{a}$ |
| | *G1* | $5.38 \pm 0.03^{e}$ | $8.00 \pm 0.43^{d}$ | $15.04 \pm 0.65^{f}$ | $8.67 \pm 0.31^{de}$ | $2.76 \pm 0.36^{fg}$ |
| | *H1* | $8.60 \pm 0.05^{c}$ | $12.75 \pm 0.73^{bc}$ | $21.04 \pm 0.53^{cd}$ | $11.84 \pm 0.74^{c}$ | $3.82 \pm 0.31^{cde}$ |
| | *I1* | $5.44 \pm 0.16^{e}$ | $7.27 \pm 0.87^{d}$ | $16.93 \pm 0.50^{e}$ | $9.20 \pm 0.34^{d}$ | $2.64 \pm 0.39^{g}$ |
| | *Golden Delicious* | $4.66 \pm 0.22^{fg}$ | $6.14 \pm 0.30^{d}$ | $11.50 \pm 0.43^{h}$ | $6.92 \pm 0.74^{f}$ | $4.46 \pm 0.50^{bc}$ |
| | *Red Delicious* | $4.25 \pm 0.59^{gh}$ | $6.63 \pm 0.34^{d}$ | $12.96 \pm 0.25^{g}$ | $7.91 \pm 0.28^{ef}$ | $4.32 \pm 0.36^{cd}$ |
| | *Granny smith* | $5.11 \pm 0.17^{ef}$ | $7.63 \pm 0.61^{d}$ | $13.57 \pm 0.79^{g}$ | $8.74 \pm 0.23^{de}$ | $3.50 \pm 0.64^{def}$ |
| | *Royal Gala* | $3.77 \pm 0.25^{h}$ | $6.98 \pm 0.59^{d}$ | $12.77 \pm 0.12^{gh}$ | $7.86 \pm 0.22^{ef}$ | $5.17 \pm 0.37^{ab}$ |
| **Peels** | *A2* | $14.16 \pm 0.13^{fg}$ | $23.54 \pm 1.45$ | $37.89 \pm 0.79^{gh}$ | $21.33 \pm 1.74^{d}$ | $5.05 \pm 0.25^{e}$ |
| | *B1* | $20.33 \pm 0.05^{a}$ | $34.26 \pm 0.32$ | $53.69 \pm 1.63^{e}$ | $31.59 \pm 0.56^{b}$ | $5.93 \pm 0.30^{cd}$ |
| | *C1* | $17.37 \pm 0.58^{d}$ | $30.02 \pm 0.83$ | $41.32 \pm 0.36^{f}$ | $23.03 \pm 0.67^{cd}$ | $6.76 \pm 0.24^{b}$ |
| | *D2* | $18.11 \pm 0.32^{c}$ | $33.24 \pm 0.33$ | $49.53 \pm 1.14^{d}$ | $30.45 \pm 1.73^{b}$ | $5.01 \pm 0.13^{e}$ |
| | *E2* | $19.41 \pm 0.16^{b}$ | $32.31 \pm 0.74$ | $56.30 \pm 2.03^{b}$ | $34.74 \pm 0.77^{a}$ | $4.51 \pm 0.26^{ef}$ |
| | *F1* | $20.31 \pm 0.10^{a}$ | $34.05 \pm 0.14$ | $62.75 \pm 0.54^{a}$ | $37.66 \pm 1.08^{a}$ | $5.85 \pm 0.18^{d}$ |
| | *G1* | $16.73 \pm 0.23^{e}$ | $23.22 \pm 1.70$ | $38.80 \pm 0.41^{g}$ | $22.16 \pm 1.49^{d}$ | $4.61 \pm 0.30^{ef}$ |
| | *H1* | $20.11 \pm 0.04^{a}$ | $31.77 \pm 1.06$ | $54.31 \pm 0.44^{bc}$ | $31.61 \pm 0.94^{b}$ | $6.34 \pm 0.50^{bcd}$ |
| | *I1* | $17.69 \pm 0.13^{cd}$ | $28.26 \pm 1.73$ | $49.71 \pm 0.49^{d}$ | $29.27 \pm 1.20^{b}$ | $4.11 \pm 0.39^{f}$ |
| | *Golden Delicious* | $11.75 \pm 0.13^{h}$ | $18.02 \pm 0.68$ | $25.26 \pm 0.70^{j}$ | $14.71 \pm 0.68^{e}$ | $4.50 \pm 0.53^{ef}$ |
| | *Red Delicious* | $19.01 \pm 0.10^{b}$ | $31.67 \pm 1.55$ | $45.03 \pm 0.43^{e}$ | $25.37 \pm 1.35^{c}$ | $6.12 \pm 0.30^{bcd}$ |
| | *Granny smith* | $14.55 \pm 0.24^{f}$ | $23.96 \pm 0.62$ | $35.51 \pm 0.47^{h}$ | $20.05 \pm 1.74^{d}$ | $8.16 \pm 0.24^{a}$ |
| | *Royal Gala* | $13.60 \pm 0.09^{g}$ | $19.98 \pm 0.78$ | $29.31 \pm 1.56^{i}$ | $16.20 \pm 1.51^{e}$ | $6.53 \pm 0.44^{bc}$ |

[*] Values expressed are means ± S.D. of three parallel measurements. TE: Trolox equivalent; EDTAE: EDTA equivalent. Different letters indicated significant differences in each group (pulp or peels) ($p < 0.05$).

Table 3. Enzyme inhibitory effects of the apple samples

| | Samples | AChE inhibition (mgGALAE/g) | BChE inhibition (mgGALAE/g) | Tyrosinase (mgKAE/g) | Amylase (mgACAE/g) | Glucosidase (mgACAE/g) |
|---|---|---|---|---|---|---|
| **Pulp** | *A2* | 1.07±0.02$^{ef*}$ | 0.49±0.01$^{cd}$ | 25.50±1.36$^{g}$ | 5.74±0.35$^{ab}$ | 21.38±0.57$^{d}$ |
| | *B1* | 0.97±0.05$^{g}$ | 0.55±0.05$^{bcd}$ | 25.84±0.36$^{fg}$ | 5.02±0.41$^{cde}$ | 34.26±0.16$^{a}$ |
| | *C1* | 1.00±0.04$^{fg}$ | 0.46±0.05$^{d}$ | 26.57±0.83$^{defg}$ | 5.94±0.26$^{ab}$ | 33.03±0.89$^{ab}$ |
| | *D2* | 1.24±0.04$^{ab}$ | 0.60±0.09$^{ab}$ | 27.01±0.26$^{bcde}$ | 4.76±0.13$^{def}$ | 34.44±0.02$^{a}$ |
| | *E2* | 1.18±0.01$^{bc}$ | 0.68±0.01$^{a}$ | 26.71±0.06$^{cdef}$ | 5.71±0.19$^{abc}$ | 34.50±0.01$^{a}$ |
| | *F1* | 1.08±0.04$^{de}$ | 0.60±0.01$^{ab}$ | 29.25±0.59$^{a}$ | 6.34±0.18$^{a}$ | 32.81±0.69$^{ab}$ |
| | *G1* | 1.14±0.03$^{cd}$ | 0.59±0.03$^{abc}$ | 26.86±0.06$^{cdef}$ | 5.64±0.33$^{abc}$ | 31.26±2.12$^{b}$ |
| | *H1* | 1.11±0.01$^{cde}$ | 0.52±0.10$^{bcd}$ | 28.13±0.48$^{ab}$ | 4.61±0.32$^{def}$ | 31.50±1.58$^{b}$ |
| | *I1* | 1.26±0.01$^{a}$ | 0.59±0.03$^{abc}$ | 28.08±0.34$^{b}$ | 5.25±0.10$^{bcd}$ | 34.26±0.10$^{a}$ |
| | *Golden Delicious* | 0.98±0.05$^{g}$ | 0.59±0.01$^{abc}$ | 26.04±0.75$^{efg}$ | 4.35±0.36$^{ef}$ | 33.22±0.63$^{ab}$ |
| | *Red Delicious* | 1.22±0.05$^{ab}$ | 0.49±0.05$^{cd}$ | 27.73±0.28$^{bcd}$ | 4.81±0.11$^{def}$ | 25.26±2.87$^{c}$ |
| | *Granny smith* | 1.29±0.02$^{a}$ | 0.59±0.01$^{abc}$ | 27.28±0.35$^{bcd}$ | 4.28±0.41$^{f}$ | 32.44±0.77$^{ab}$ |
| | *Royal Gala* | 1.14±0.04$^{cde}$ | 0.51±0.10$^{bcd}$ | 27.79±0.26$^{bc}$ | 4.49±0.26$^{ef}$ | 24.44±1.89$^{c}$ |
| **Peels** | *A2* | 1.22±0.05$^{ab}$ | 0.56±0.01$^{bcde}$ | 27.60±0.16$^{d}$ | 6.99±0.26$^{a}$ | 34.11±0.01$^{a}$ |
| | *B1* | 1.20±0.04$^{abc}$ | 0.58±0.05$^{abcd}$ | 28.59±0.23$^{bc}$ | 6.25±0.21$^{abc}$ | 34.38±0.05$^{a}$ |
| | *C1* | 1.13±0.03$^{de}$ | 0.64±0.01$^{a}$ | 28.48±0.05$^{c}$ | 6.48±0.57$^{ab}$ | 34.11±0.04$^{a}$ |
| | *D2* | 1.23±0.01$^{a}$ | 0.59±0.02$^{abc}$ | 28.80±0.23$^{abc}$ | 6.68±0.39$^{ab}$ | 34.50±0.06$^{a}$ |
| | *E2* | 1.16±0.02$^{bcde}$ | 0.59±0.01$^{abc}$ | 29.08±0.41$^{abc}$ | 6.31±0.51$^{abc}$ | 34.45±0.02$^{a}$ |
| | *F1* | 1.20±0.02$^{abc}$ | 0.61±0.05$^{abc}$ | 29.27±0.16$^{a}$ | 6.46±0.23$^{ab}$ | 34.47±0.07$^{a}$ |
| | *G1* | 1.19±0.01$^{abcd}$ | 0.58±0.01$^{abcd}$ | 29.35±0.12$^{a}$ | 5.24±0.51$^{d}$ | 34.39±0.02$^{a}$ |
| | *H1* | 1.15±0.05$^{cde}$ | 0.64±0.01$^{a}$ | 29.19±0.30$^{ab}$ | 5.39±0.14$^{cd}$ | 34.30±0.03$^{a}$ |
| | *I1* | 1.21±0.01$^{abc}$ | 0.51±0.06$^{de}$ | 28.77±0.18$^{abc}$ | 6.19±0.32$^{abc}$ | 34.49±0.06$^{a}$ |
| | *Golden Delicious* | 1.19±0.02$^{abcd}$ | 0.54±0.06$^{cde}$ | 27.70±0.31$^{d}$ | 6.94±0.16$^{a}$ | 34.34±0.04$^{a}$ |
| | *Red Delicious* | 1.11±0.02$^{e}$ | 0.63±0.04$^{ab}$ | 27.20±0.77$^{d}$ | 6.00±0.18$^{bcd}$ | 33.07±0.64$^{a}$ |
| | *Granny smith* | 1.13±0.03$^{e}$ | 0.34±0.03$^{f}$ | 25.14±0.48$^{e}$ | 6.71±0.32$^{ab}$ | 30.72±1.68$^{b}$ |
| | *Royal Gala* | 0.93±0.03$^{f}$ | 0.49±0.02$^{e}$ | 27.06±0.08$^{d}$ | 6.14±0.63$^{abcd}$ | 27.60±1.88$^{c}$ |

[*] Values expressed are means ± S.D. of three parallel measurements. GALAE: Galatamine equivalent; KAE: Kojic acid equivalent; ACAE: Acarbose equivalent. Different letters indicated significant differences in each group (pulp or peels) ($p < 0.05$).

Scheme 1. Structure of main triterpene acid of apple

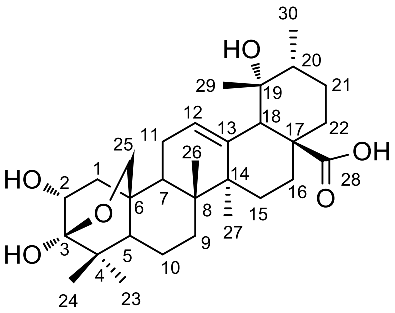


Pomaceic acid

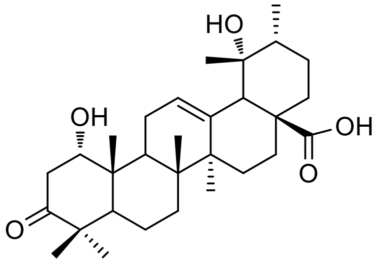


Annurcoic acid

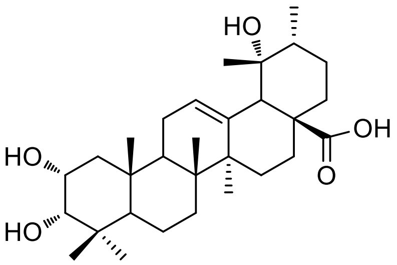


Euscaphyc acid

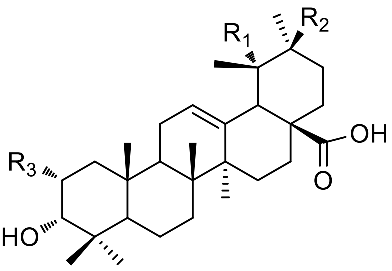


$R_1$ OH, $R_2$ H, $R_3$ H, Pomolic acid
$R_1$ H, $R_2$ H, $R_3$ OH Corosolic acid

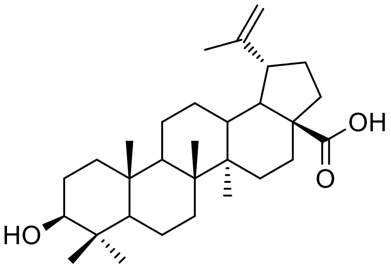


Betulinic acid

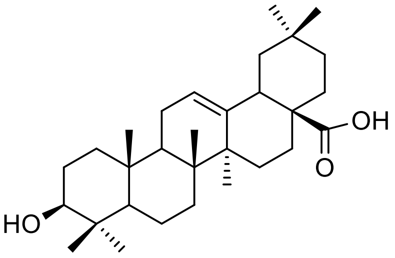


Oleanolic acid

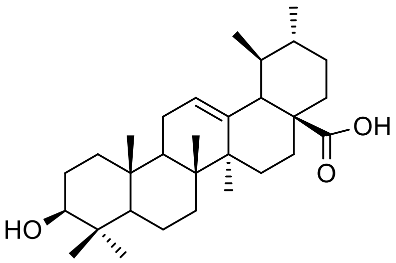


Ursolic acid

Figure 1. MS Spectra of Pomaceic acid.

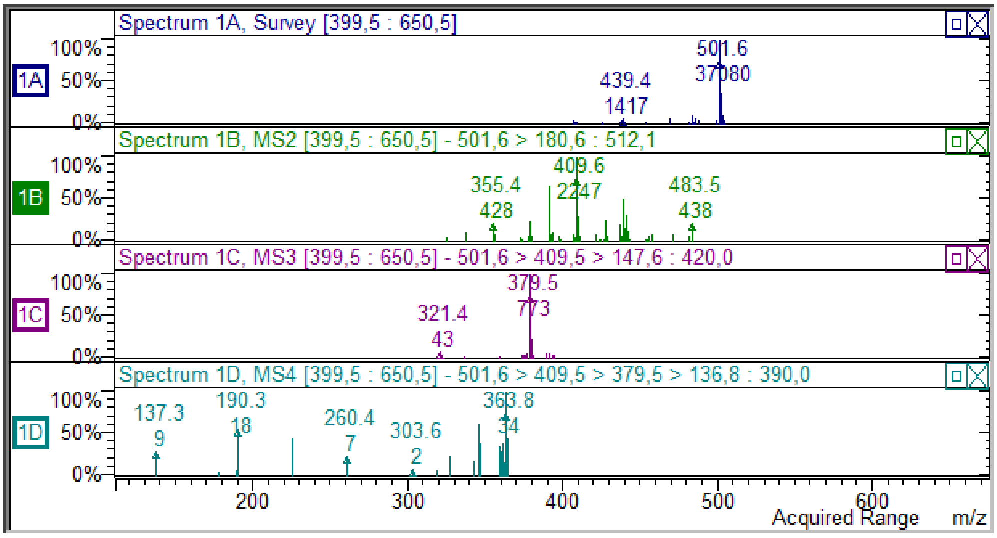


Figure 2. MS Spectra of Euscaphyc acid

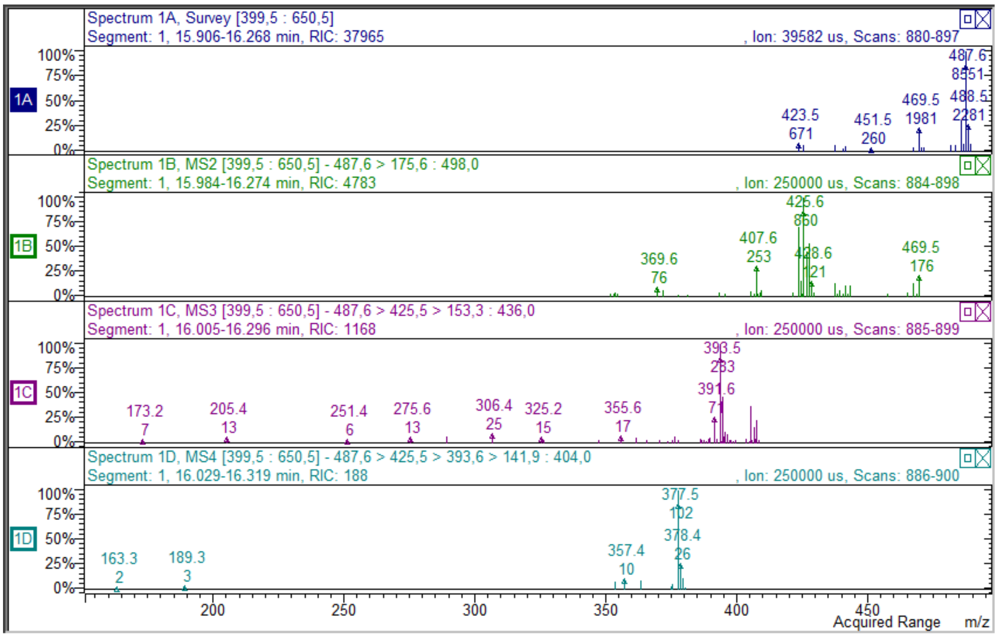

Figure 3. MS Spectra of Annurcoic acid

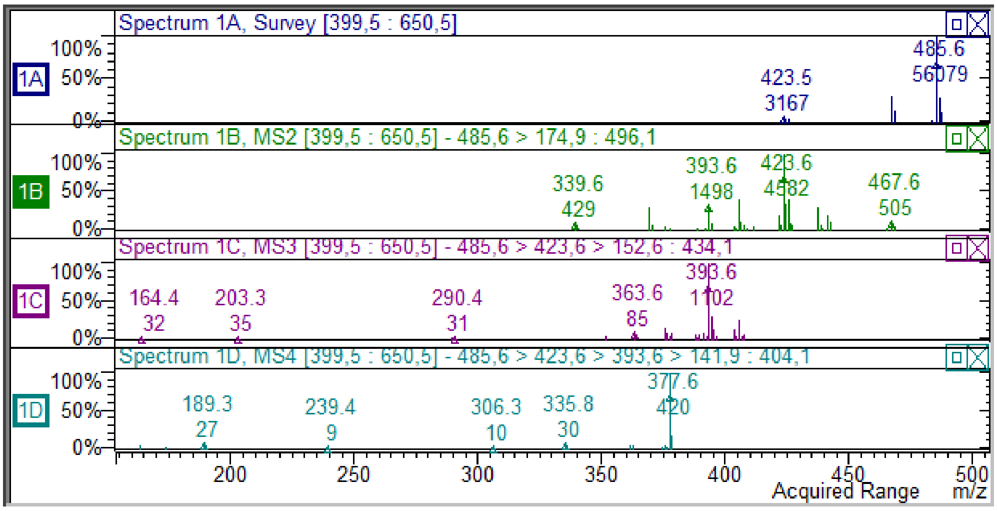


Figure 4. MS Spectra of pomolic acid.

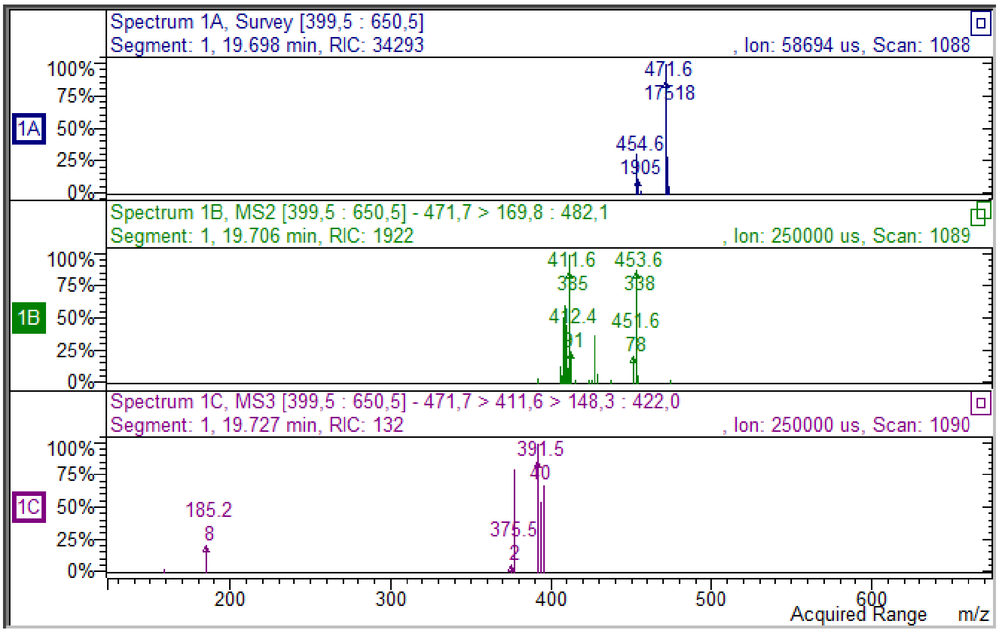

Figure 5. MS Spectra of maslinic acid

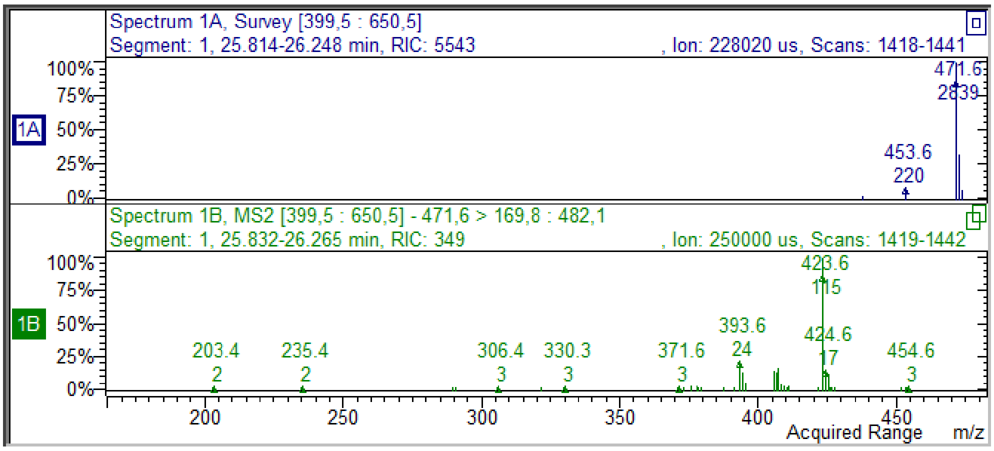


Figure 6. MS Spectra of corosolic acid

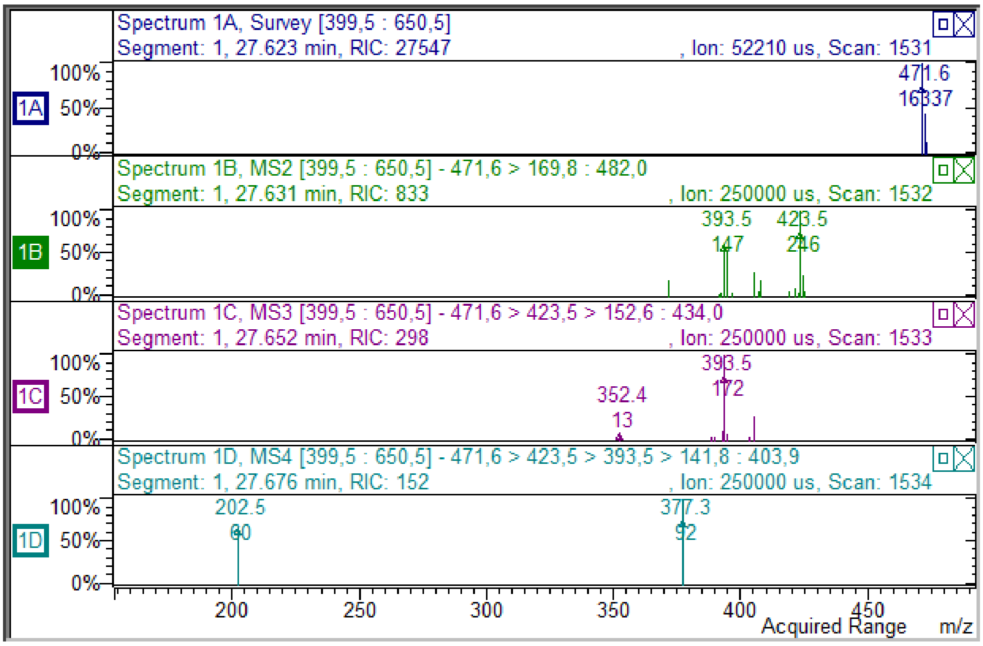

Figure 7. MS Spectra of betulinic acid

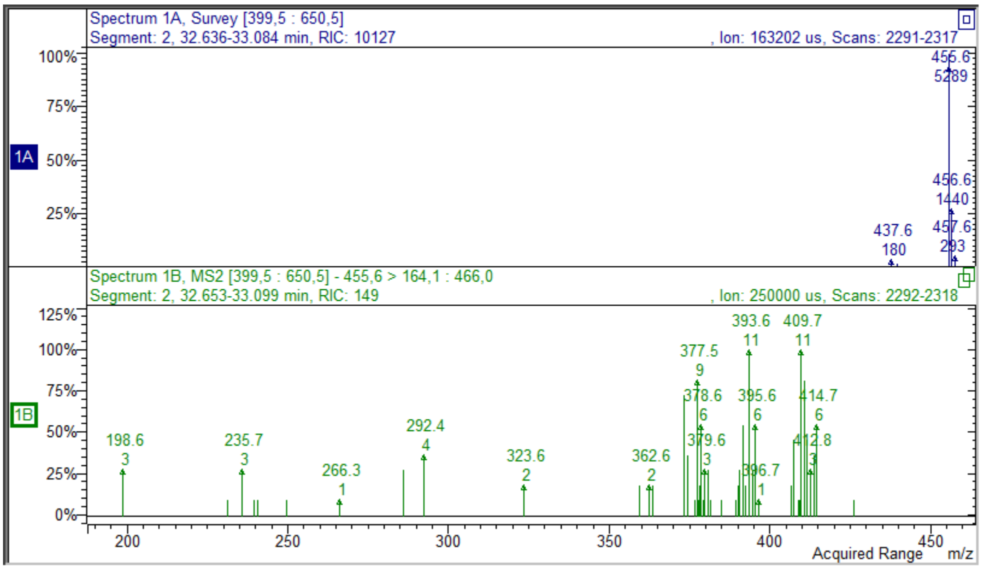


Figure 8. MS Spectra of oleanolic acid

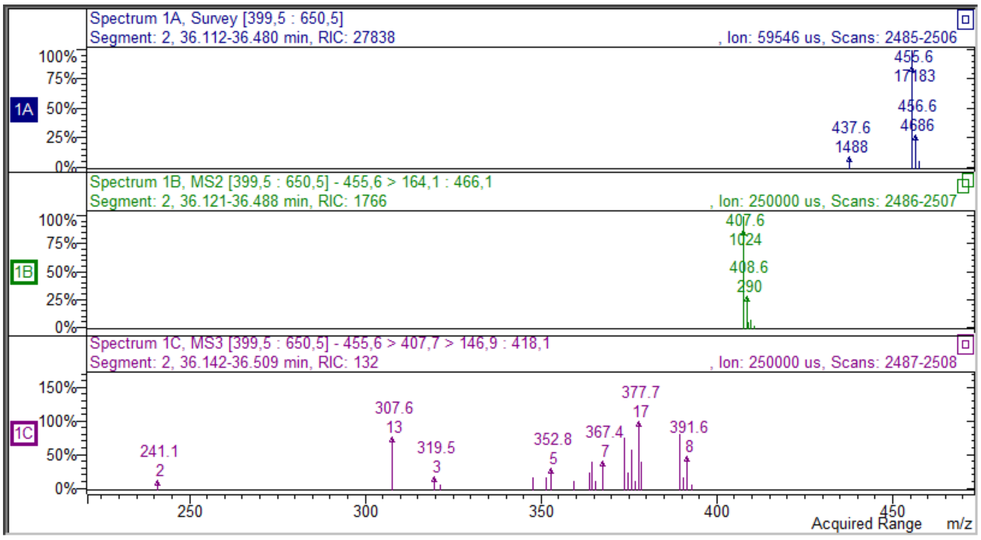

Figure 9. MS Spectra of ursolic acid

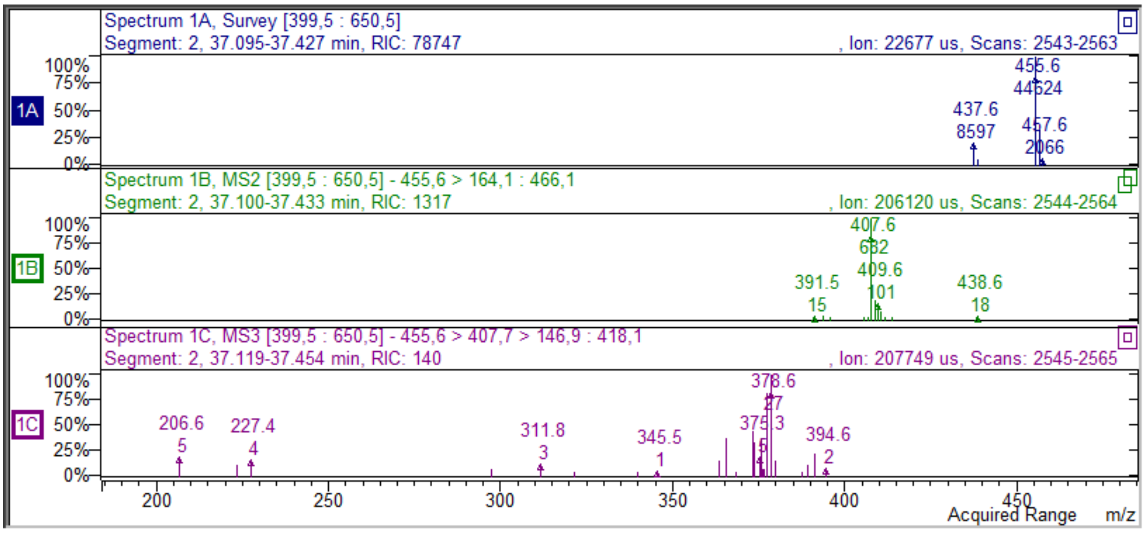


Figure 10. Representative chromatogram of apple peels; the principal classes of phytoconstituents are highlighted; numbers of the identified metabolites are those reported in Table 1.

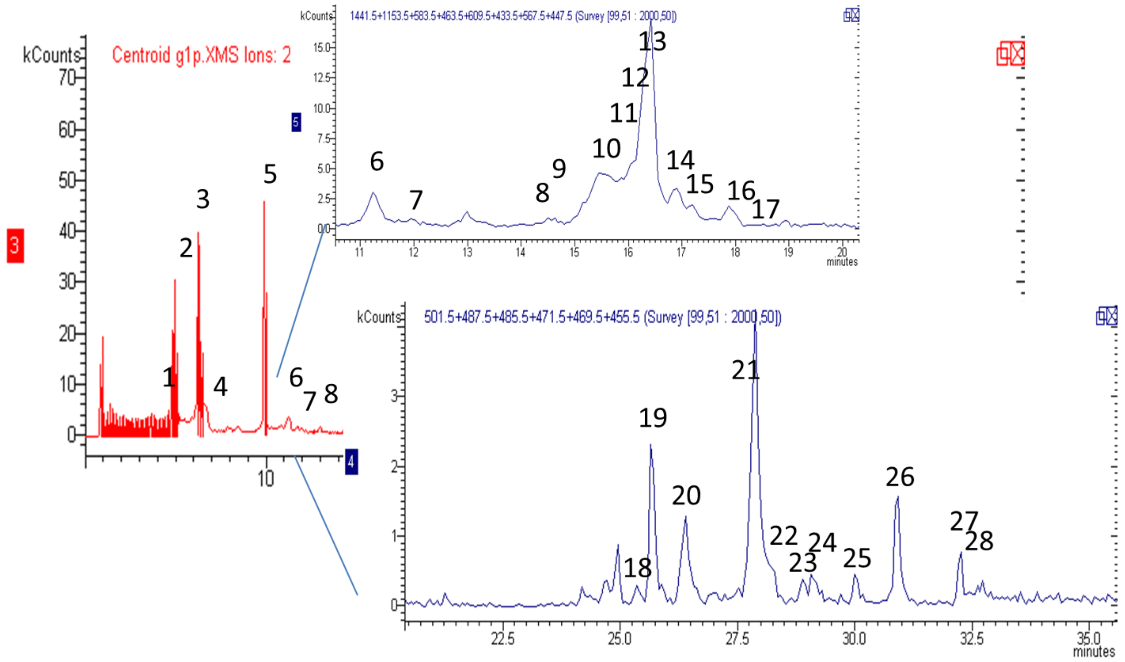

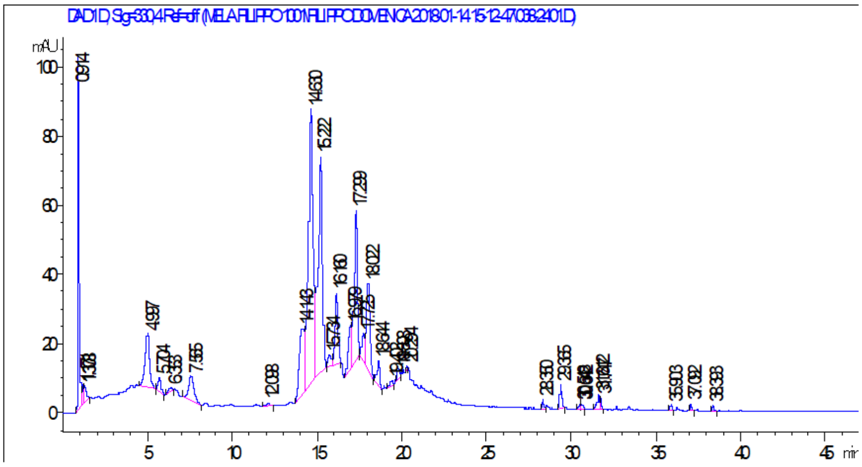


Peels sample E1 della Rosa

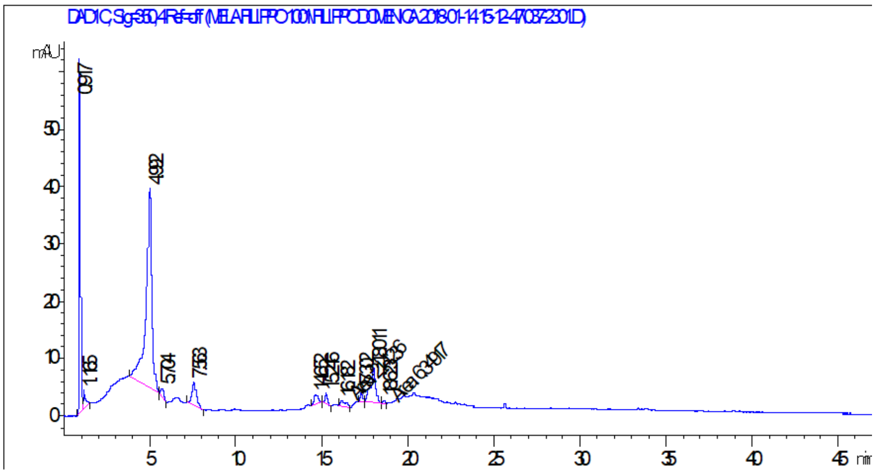


Pulp sample E1 della Rosa
